# Supplementary material for: Mature IgDlow/- B cells maintain tolerance by promoting regulatory T cell homeostasis
Source: Nat Commun. 2019 Jan 14;10:190. doi: 10.1038/s41467-018-08122-9 (PMC6331566; doi:10.1038/s41467-018-08122-9)
Supplement: Supplementary file 1 — Supplementary Information [file 41467_2018_8122_MOESM1_ESM.pdf]

Supplementary Materials

Mature IgD$^{low/-}$ B Cells Maintain Tolerance by Promoting Regulatory T Cell Homeostasis

Ray, et al.

Supplementary Figure 1

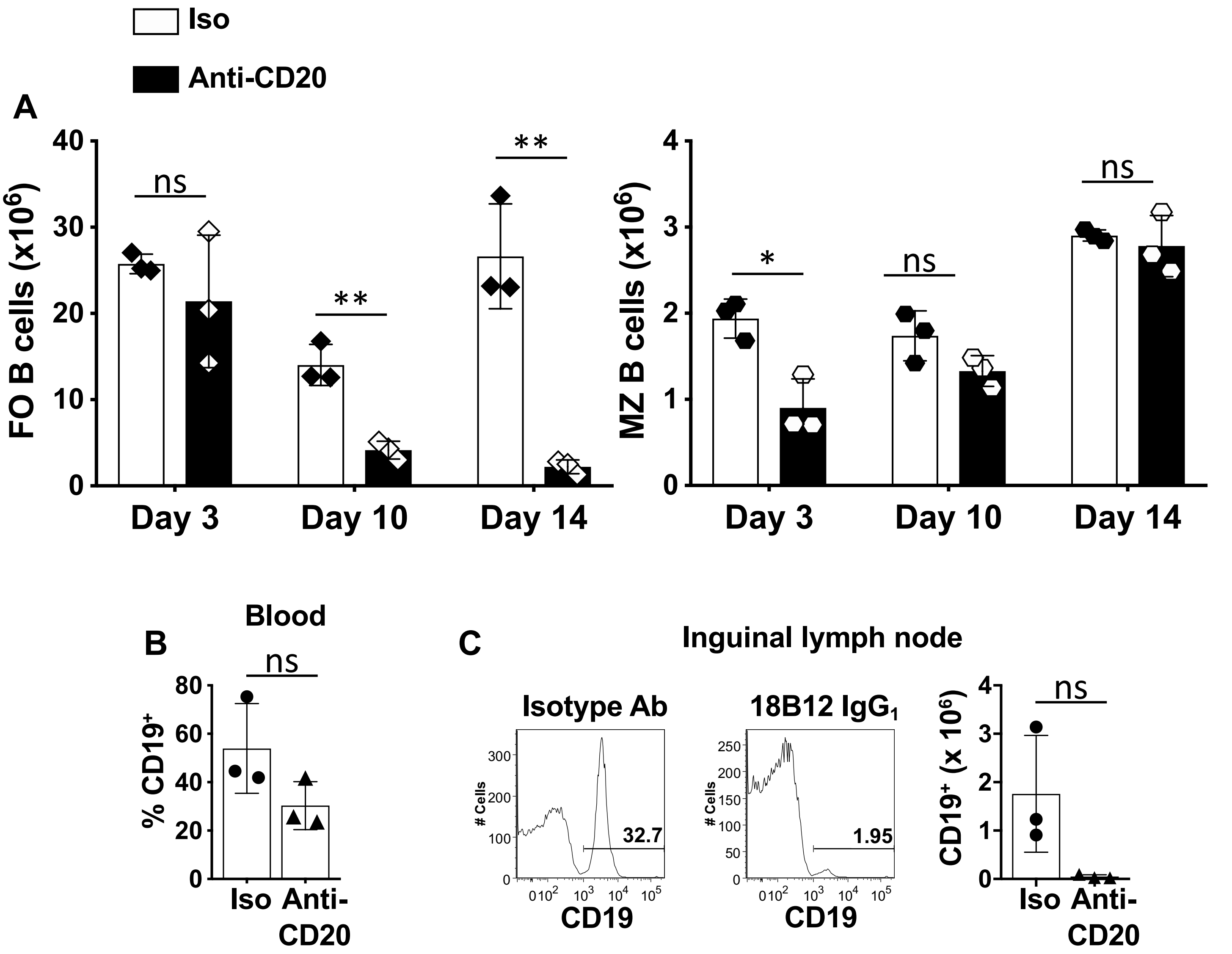

Supplemental Figure 1. B cell depletion in the spleen, blood and inguinal lymph nodes with anti-CD20 IgG_1. B10.PL mice were i.v. administered anti-CD20 (18B12IgG1) or its isotype control (2B8msIgG1) (250 mg). Three (A), 10 (A) or 14 (A-C) days later the absolute number of total B cells ($CD19^+$) in the spleen (A), peripheral blood (B) and inguinal lymph nodes (C) was determined by flow cytometry. Individual data points (mice) are shown superimposed upon the mean ± SEM. C) The left two histograms show representative flow cytometry gating. *p<0.05; **p<0.01; ns = not significant.

Supplementary Figure 2

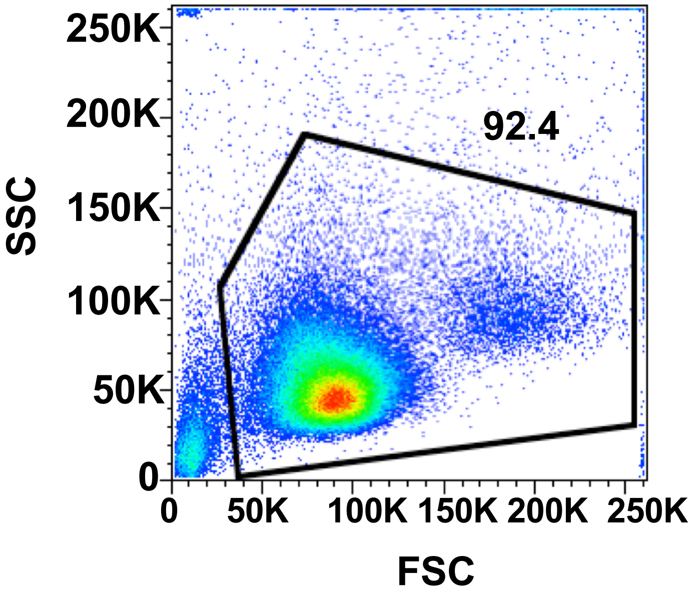

Supplemental Figure 2. Representative gating strategy for mouse CD4$^+$ T cells and Treg. Single cell suspensions from the spleen were stained for CD4 and Foxp3. The cells were gated for lymphocytes (left panel) and subsequently analyzed for CD4 and Foxp3 expression (right panel). CD4$^+$Foxp3$^+$ Treg are located in the upper right gate. This gating strategy is for Fig. 1D, 3A, 3B, 3D, 3E, 3G and 5B-D.

Supplementary Figure 3

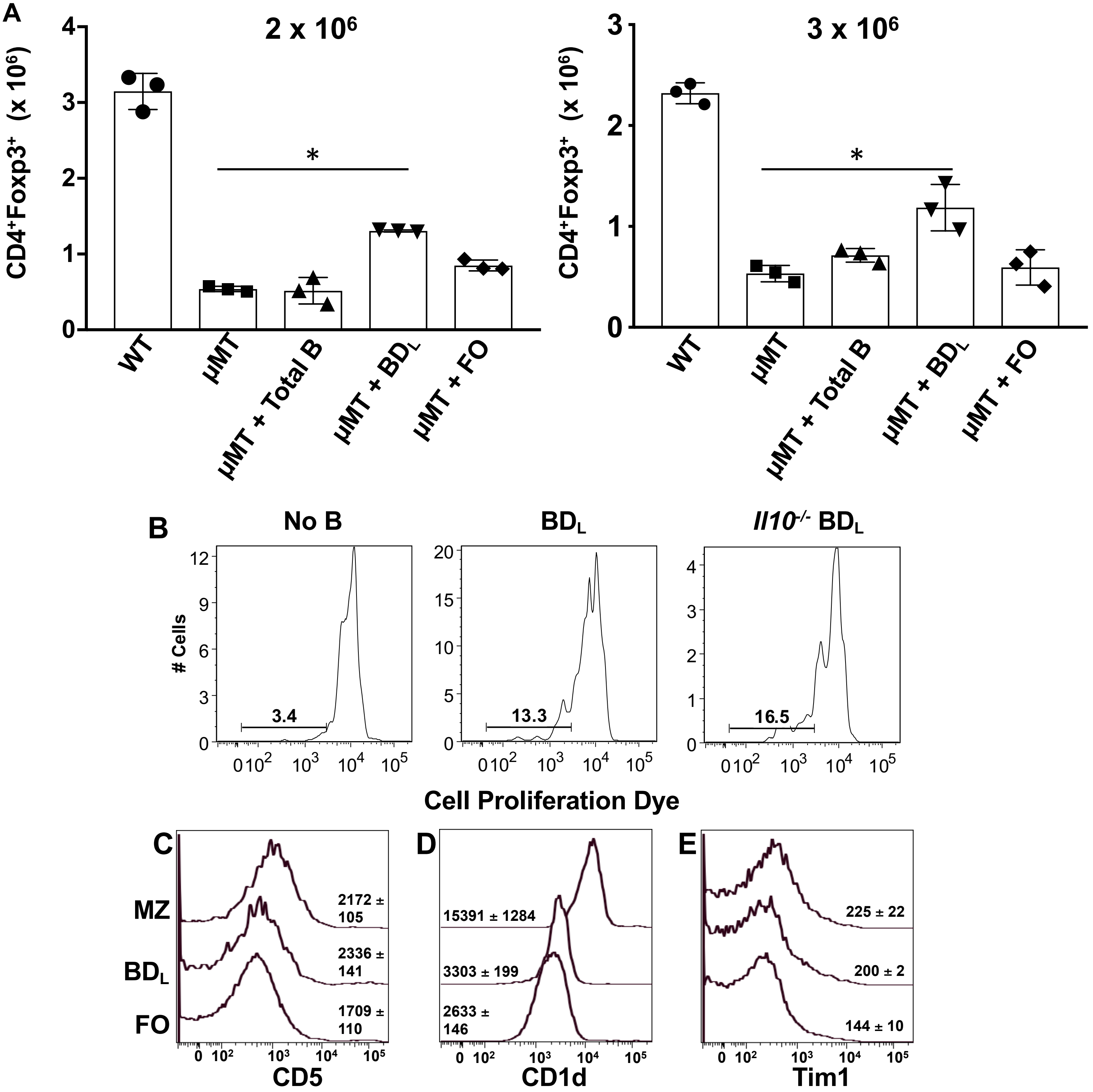

Supplemental Figure 3. BD_L induce Treg proliferation in a dose-responsive manner independent of IL-10. B10.PLμMT mice were reconstituted with 2 x 10^6 (A, left panel) or 3 x 10^6 (B, right panel) FACS purified total, BD_L or FO B cells and the absolute number of splenic Treg was determined by flow cytometry 10 days later. WT and μMT mice received PBS. Individual data points (mice) are shown superimposed upon the mean ± SEM. *p<0.05. B) Splenic Treg ($CD4^+Foxp3^{EGFP}$) were sorted and labeled with cell proliferation dye and were i.v. transferred (0.2 x 10^6) into μMT mice with WT or *Il10*$^{-/-}$ FACS purified BD_L (5 x 10^6) or alone. Seven days later proliferation of the labeled $EGFP^+$ Treg in the spleen was determined by flow cytometry. WT C57BL/6 mice were used to assess expression levels of CD5 (C), CD1d (D) and Tim1 (E) on BD_L by flow cytometry.

Supplementary Figure 4

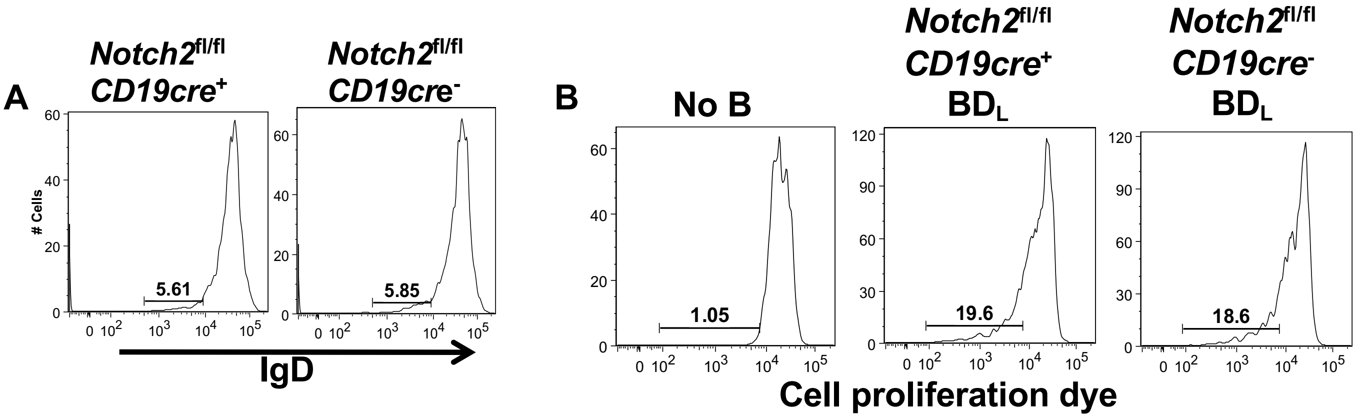

Supplemental Figure 4. BD_L are not from the MZ B cell lineage. A) The presence of BD_L was determined in the spleen of *Notch2*$^{fl/fl}$*CD19cre*$^+$ and *Notch2*$^{fl/fl}$*CD19cre*$^-$ mice by flow cytometry. Splenic Treg ($CD4^+Foxp3^{EGFP}$) were FACS purified and labeled with cell proliferation dye and were i.v. transferred (0.2 x 10^6) into μMT mice with WT, *Notch2*$^{fl/fl}$*CD19cre*$^+$ or *Notch2*$^{fl/fl}$*CD19cre*$^-$ FACS purified BD_L (5 x 10^6) or alone. Seven days later proliferation of the labeled $EGFP^+$ Treg in the spleen was determined by flow cytometry.

Supplementary Figure 5

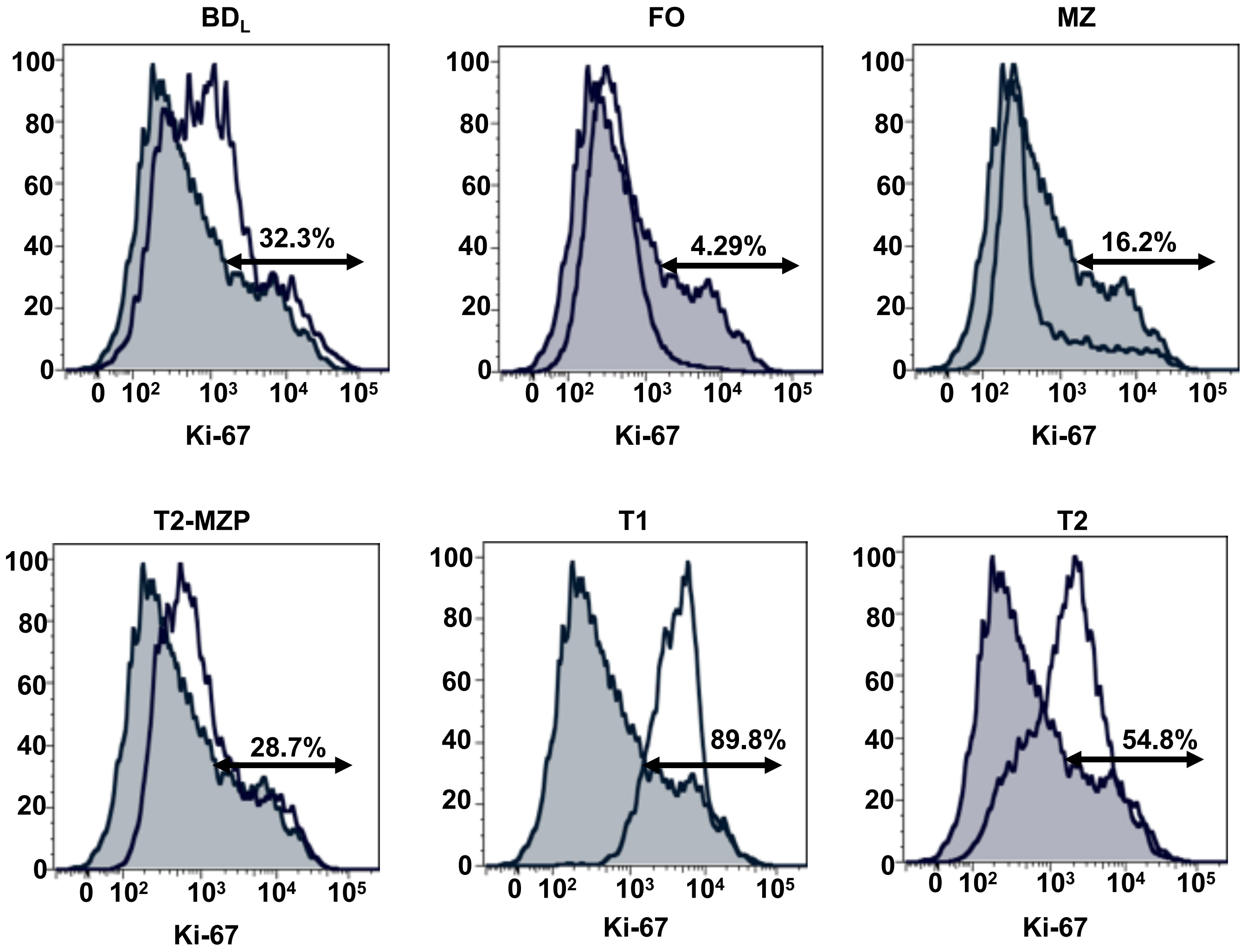

Supplemental Figure 5. Representative gating strategy for Ki-67 analysis. Single cell suspensions from the spleen were stained for B cell markers and gated as for Fig. 2A for BD$_L$, FO, MZ, T2-MZP, T1 and T2 subsets. The gating control is a single color Ki-67 stain of total splenocytes in which the negative and positive populations are discernable. The positive control gating is shown by the arrow. This gating strategy is for Fig. 4F.

Supplementary Figure 6

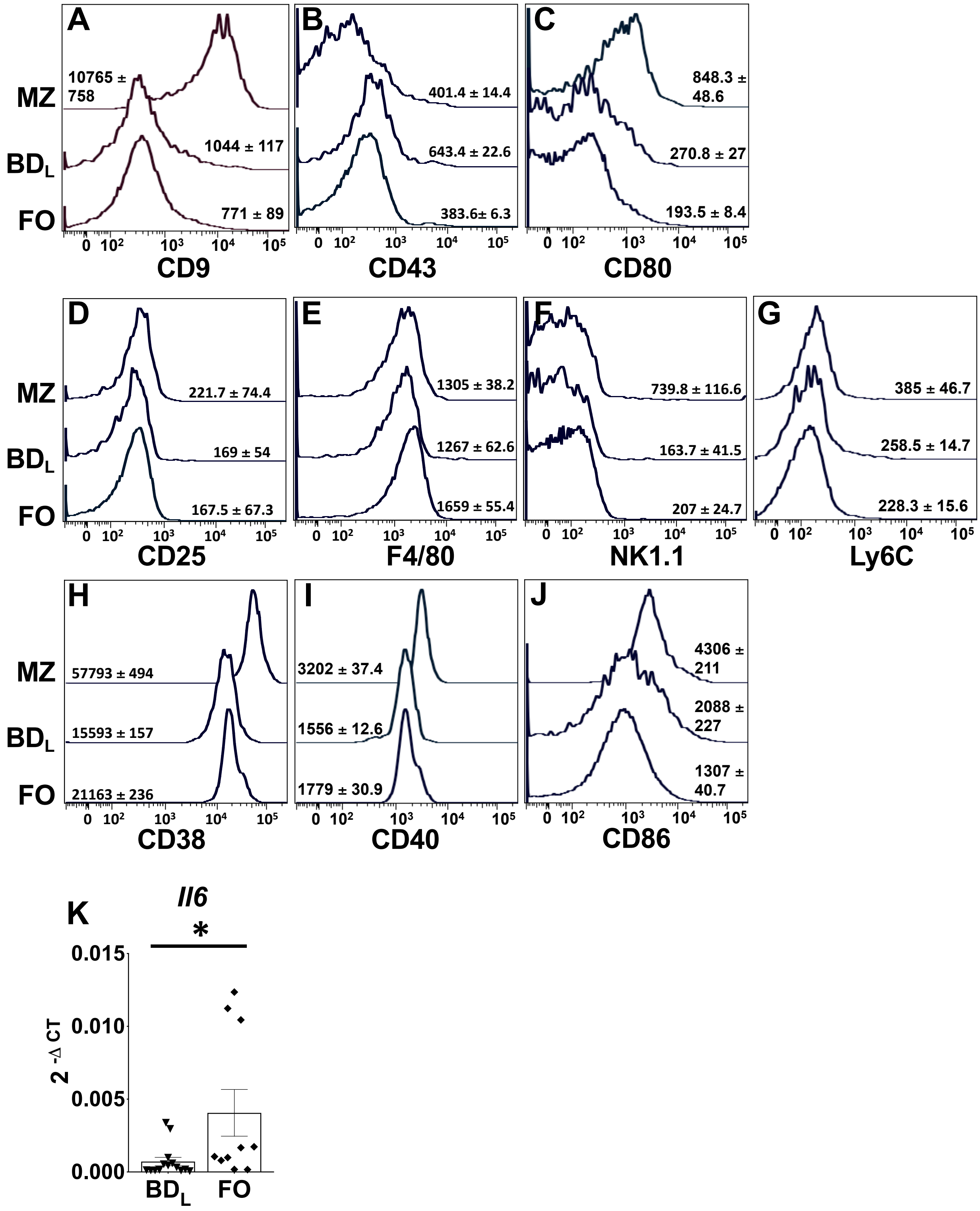

Supplementary Figure 6. Phenotypic comparisons of FO, BD_L and MZ B cells. A-J) WT C57BL/6 mice were used to assess cell surface expression levels of the indicated proteins on FO, BD_L and MZ B cells by flow cytometry. The mean fluorescence intensity ± SEM is shown from 4-9 mice. K) BD_L and FO B cells were FACS purified and following cDNA generation qPCR was utilized to assess relative levels of *Il6* mRNA expression. The *Il6* primers used are: Forward-5'-TAGTCCTTCCTACCCCAATTTCC-3`; Reverse-5`-TTGGTCCTTAGCCACTCCTTC-3`. Individual data points are shown superimposed upon the mean ± SEM. *p<0.05.

Supplementary Figure 7

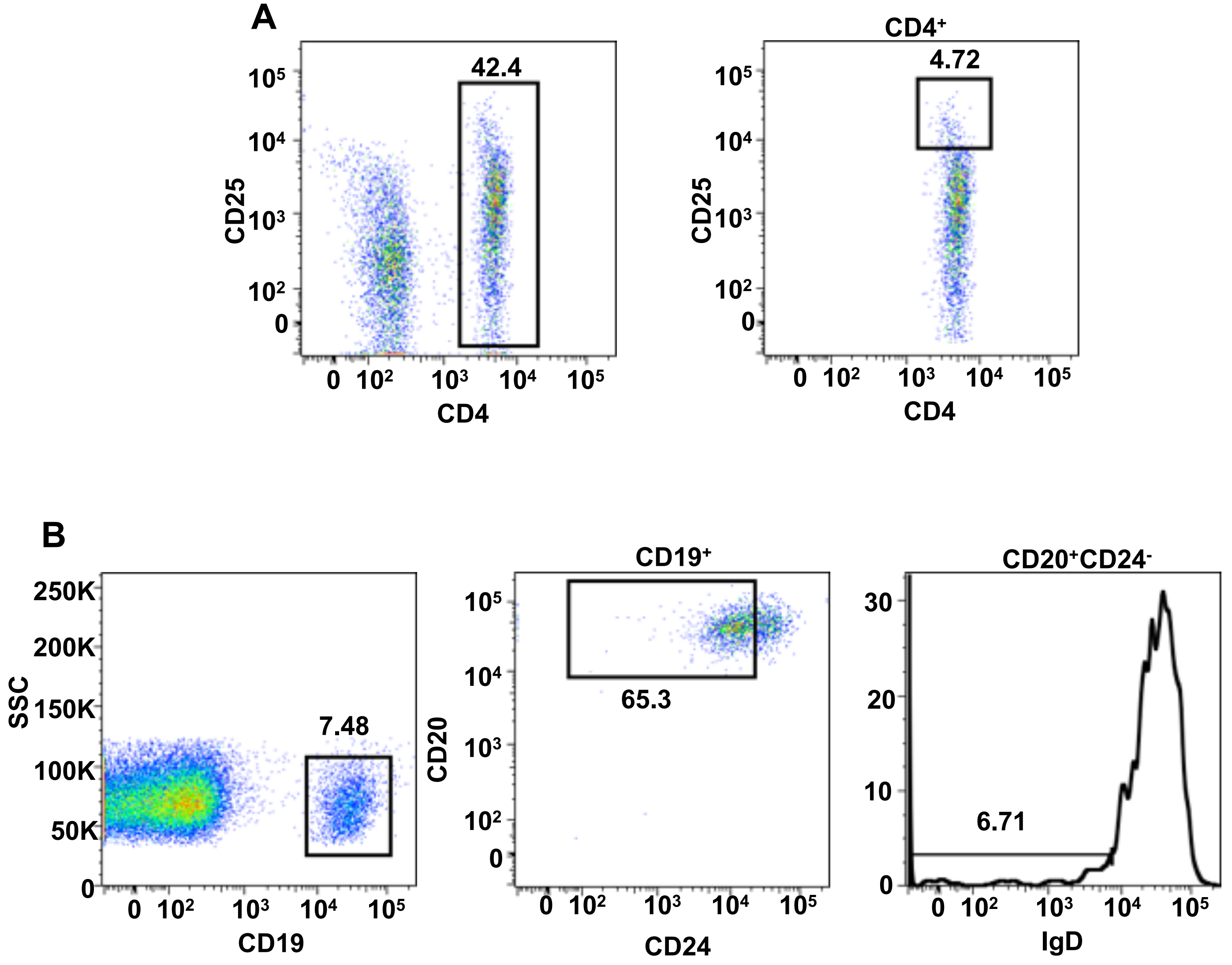

Supplemental Figure 7. Representative gating strategy for human Treg and IgD$^{low/-}$ and IgD^{hi} B cells. A) Single cell suspensions from human spleen were stained for CD4 and CD25. CD4$^+$ T cells were gated for CD25^{hi} Treg. This gating strategy is for Fig. 9D,F. B) Human peripheral blood was stained for CD19, CD20, CD24 and IgD. CD19$^+$ B cells were analyzed for expression of CD20 and CD24. CD20$^+$CD24$^-$ mature B cells were analyzed for expression of IgD. The percentage of cells in the indicated gates is provided.

Supplementary Table 1. Commercial Antibodies Utilized

Antibody[1]	Specificity[2]	Source[3]	Catalog[4]	Dilution[5]
B220-PE/CF594	Mouse	BD Biosciences	562290	1:100
GITRL-Alexa Fluor 647	Mouse	BD Biosciences	563542	1:100
GITRL-Purified	Mouse	Biolegend	120302	1:100
B220-Alexa Fluor 700	Mouse/Human	Biolegend	103232	1:100
IgM-PE Dazzle 594	Mouse	Biolegend	406529	1:100
IgD-PE	Mouse	Biolegend	405705	1:100
IgD-Alexa Fluor 488	Mouse	Biolegend	405717	1:100
CD45.1-Alexa Fluor 647	Mouse	Biolegend	110720	1:100
Ki-67-APC	Mouse	Biolegend	652405	1:200
CD36-APC	Mouse	Biolegend	102611	1:100
CD5-PE/Cy5	Mouse	Biolegend	100609	1:100
CD20-APC	Mouse	Biolegend	150411	1:100
CD365 (Tim-1)-PE	Mouse	Biolegend	119505	1:100
CD1d-APC	Mouse	Biolegend	123521	1:100
CD9-Alexa Fluor 647	Mouse	Biolegend	124809	1:100
CD3-Brilliant Violet 421	Mouse	Biolegend	100228	1:100
CD43-PE	Mouse	Biolegend	121207	1:100
CD38-AF647	Mouse	Biolegend	102716	1:100
F4/80-PE/Cy5	Mouse	Biolegend	123112	1:100
NK1.1-APC	Mouse	Biolegend	108709	1:100
CD40-PE	Mouse	Biolegend	102805	1:100
CD86-PE	Mouse	Biolegend	105007	1:100
Ly6C-APC	Mouse	Biolegend	105007	1:100
TCR-β-FITC	Mouse	eBioscience	11-5961-82	1:100
CD21-eFluor 450	Mouse	eBioscience	48-0212-82	1:100

CD23-PE/Cy7	Mouse	eBioscience	25-0232-82	1:100
CD93-PE	Mouse	eBioscience	12-5892-82	1:100
CD80-PE/Cy5	Mouse	eBioscience	15-0801-82	1:100
IgD-FITC	Mouse	eBioscience	11-5993-85	1:100
IgM-APC	Mouse	eBioscience	17-5790-82	1:100
CD4-FITC	Mouse	eBioscience	11-0041-85	1:100
Foxp3-PE	Mouse	eBioscience	12-5773-80	1:100
CD209b (SIGN-R1)-APC	Mouse	eBioscience	17-2093-80	1:100
CD25-APC	Mouse	Caltag	RM6005	1:100
CD4-APC/Cy7	Human	Biolegend	317417	1:100
CD19-PerCP/Cy5.5	Human	Biolegend	302229	1:100
CD20-AF700	Human	Biolegend	302322	1:100
CD24-PE/Cy7	Human	Biolegend	311119	1:100
IgD-Brilliant Violet 421	Human	Biolegend	348225	1:100
CD25-PE	Human	Biolegend	356103	1:100
CD3	Human	Biolegend	317303	1:100
CD28	Human	Biolegend	302913	1:100

[1]Antibody specificity and fluorochrome
[2]Species specificity of the antibody
[3]BD Bioscicences, San Diego, CA; Biolegend, San Diego, CA; eBioscience, San Diego, CA; Caltag Laboratories, Burlingame, CA
[4]Catalog number of the antibody
[5]Dilution used of the stock vial of antibody
